# Diauxic Growth at the Mesoscopic Scale

**DOI:** 10.3390/e22111280

**Published:** 2020-11-12

**Authors:** Mirosław Lachowicz, Mateusz Dȩbowski

**Affiliations:** Institute of Applied Mathematics and Mechanics, Faculty of Mathematics, Informatics and Mechanics, University of Warsaw, ul. Banacha 2, 02-097 Warsaw, Poland; mateusz.debowski@mimuw.edu.pl

**Keywords:** diauxic growth, replicator equation, mesoscopic model, integro-differential equations

## Abstract

In the present paper, we study a diauxic growth that can be generated by a class of model at the mesoscopic scale. Although the diauxic growth can be related to the macroscopic scale, similarly to the logistic scale, one may ask whether models on mesoscopic or microscopic scales may lead to such a behavior. The present paper is the first step towards the developing of the mesoscopic models that lead to a diauxic growth at the macroscopic scale. We propose various nonlinear mesoscopic models conservative or not that lead directly to some diauxic growths.

## 1. Introduction

In various processes in nature and social sciences, e.g., artificial neural networks, biology, medicine, and sociology, the logistic growth is observed in experiments. The logistic growth describes, at the macroscopic scale, the limited growth of a population. It is a typical way of modeling tumor growth—see e.g., [1,2,3] and references therein. It leads to the curve of *S*, or sigmoid, shape. In more mathematical terms a single inflection point is present. In some cases, however, a more complex behavior is observed. That was pointed out in 1949 by Monod—see [4], page 390—“*This phenomenon is characterized by a double growth cycle consisting of two exponential phases separated by a phase during which the growth rate passes through a minimum, even becoming negative in some cases*”. Monod referred such a behavior to the growth of bacterial cultures and called it—diauxie. The similar effect was hypothesized in the analysis of a role for the CDC6 protein in the entry of cells into mitosis—see [5]. Based on the experimental data in [5], a new hypothesis that CDC6 slows down the activation of inactive complexes of CDK1 and cyclin B upon mitotic entry was formulated and the corresponding mathematical model was developed. Another example is the process of DNA melting in the case when the possible base pairs of AT (or TA) and of CG (or GC) appear in two separate groups composed only of AT and CG—see Figure 7.14, page 205, in [6].

In mathematical terms, we can refer to diauxic growth, if the corresponding increasing bounded function has more than one single inflection point. The first mathematical description of such a behavior is contained in [7].

One may note that the data of total cases of COVID–19, according to Johns Hopkins University, in September 2020, show the curves with more than one inflection points in cases of various European countries, like Spain, Italy, France, Germany, and UK. On the other hand, countries like Brazil, Chile, and South Africa display curves closed to the logistic growth (with only one inflection point).

**Figure 1:**
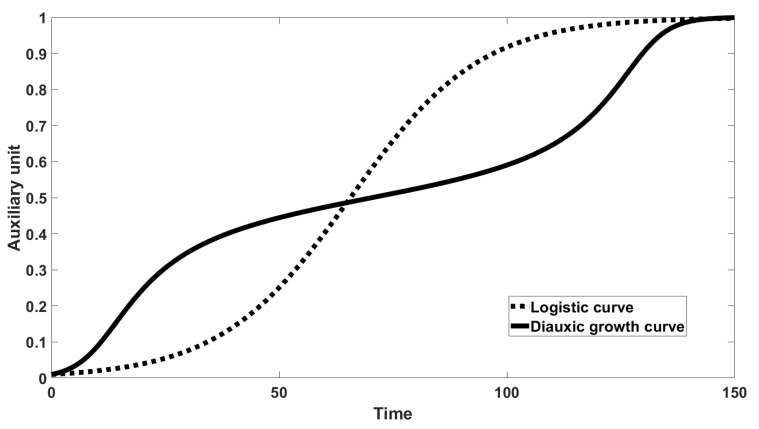
Comparison between logistic curve and diauxic growth curve.

The comparison between the logistic curve and the curve with diauxic growth is presented in Figure 1.

In the present paper, we apply

**Definition** **1.** An increasing bounded and positive-valued real function is said to have a diauxic growth if its number of inflection points is bigger than one.

Although the diauxic growth can be related, similarly to the logistic one, to the macroscopic scale one may ask whether the models on mesoscopic or microscopic scales (cf. [8]) can result in a diauxic growth. The present paper is the first step towards the developing of the mesoscopic models that lead to a diauxic growth at the macroscopic scale. We propose various nonlinear mesoscopic models, both conservative and not, which lead directly to some diauxic growths.

## 2. Replicator Equation

We consider the following replicator equations that occur in the multi-player games, see [7,9].

(1) $$\frac{d\;x}{d\;t}=x\left(1-x\right)P\left(x\right)\;,$$

where P=P(x) is a polynomial. In [7] the following polynomials were considered

(2) $$P\left(x\right)={\left(x-a\right)}^{2}+\omega \;,$$

where 0<a<1, ω>0 is a small number, and

(3) $$P\left(x\right)={\left(x-a\right)}^{2}{\left(x-b\right)}^{2}+\omega \;,$$

where 0<a<b<1 and ω is a (small) number. The former refers to three players games whereas the latter to five players games. Both are related to two strategies ↑ and ↓ in an infinitely large population. The variables *x* and 1−x are the frequencies of strategies ↑ and ↓, respectively.

Consider the following payoff matrix in the case of a 3 players (for the sake of simplicity) game

aa↑↑↑↓↓↓aaaaa↑aa1a2a3↓ab1b2b3,

where $a_{i}$, $b_{i}$, and i=1,2,3, are the corresponding payoffs. The classical way of presentation is used. For example $a_{2}$ is payoff of the “*first player*” with strategy ↑ against the other players with strategies ↑ and ↓. Again, for the sake of simplicity, we assume that the payoffs are nonnegative.

Let now μ=μ(t) and ν=ν(t) be densities of players with strategies ↑ and ↓, respectively, cf. [10]. In terms of the averages payoffs of the two strategies their dynamics is defined by the system

(4) $$\begin{array}{ccc} \frac{d\;}{d\;t}\mu & =\; & \mu \left(a_{1}\frac{\mu^{2}}{{\left(\mu +\nu \right)}^{2}}+2a_{2}\frac{\mu \;\nu }{{\left(\mu +\nu \right)}^{2}}+a_{3}\frac{\nu^{2}}{{\left(\mu +\nu \right)}^{2}}-\kappa \right)\;,\\ \frac{d\;}{d\;t}\nu & =\; & \nu \left(b_{1}\frac{\mu^{2}}{{\left(\mu +\nu \right)}^{2}}+2b_{2}\frac{\mu \;\nu }{{\left(\mu +\nu \right)}^{2}}+b_{3}\frac{\nu^{2}}{{\left(\mu +\nu \right)}^{2}}-\kappa \right)\;, \end{array}$$

where, in addition to the net growth, we consider a linear death terms with rate κ>0. We see that $x\left(t\right)=\frac{\mu (t)}{\mu (t)+\nu (t)}$ satisfies Equation (1) with

(5) $$P\left(x\right)=\hat{a}x^{2}+2\hat{b}x+\hat{x}\;,$$

where $\hat{a}=a_{3}-2a_{2}+a_{1}-b_{3}+2b_{2}-b_{1}$, $\;\hat{b}=-a_{3}+a_{2}+b_{3}-b_{2}$ and $\;\hat{x}=a_{3}-b_{3}$. We refer to these statements throughout the paper.

## 3. Mesoscopic Model

We study the time–evolution of the probability density *f*. The function f=f(t,u) is the distribution of an internal, microscopic state u∈U at time t≥0 of a (*statistical* or *test*) agent, U is a domain in $R^{d}$, d∈N={1,2,…}. Such a description then has a mesoscopic nature. An arbitrary vector u∈U can be related to a biological state, activity, opinion (e.g., political opinion), a social state of a test agent, etc.—cf. [8,11,12,13,14] and references therein. The model has therefore a wide range of possible applications in various applied sciences, such as biology, medicine, social, or political sciences.

The time evolution is defined by the general nonlinear integro–differential Boltzmann-like equation, see [14] and references therein,

(6) $$\frac{\partial }{\partial \;t}f\left(t,u\right)=Q\left[f\right]\left(t,u\right)\;,\;t>0\;,\;u\in U\;,$$

where

$$\begin{array}{c} Q\left[f\right]\left(t,u\right)=\int_{R^{d}}\left(f(t,v)\;T[f(t,\;.\;)](v,u)-f(t,u)\;T[f(t,\;.\;)](u,v)\right)\;dv\;. \end{array}$$

The nonlinear operator *Q* describes interactions between agents causing the change of state. The turning rate T[f](u,v) measures the rate for an agent with state *u* to change it into *v*. A simpler equation, with two possible states only, was studied in [15]—see also [8].

The modeling process leads to a proper choice of the turning rate.

**Case** **1.** *Let*

$$T\left[f\left(t,\;.\;\right)\right]\left(u,v\right)=\beta \left(u,v\right)f^{\gamma }\left(t,v\right)\;,\;u,v\in U\;,$$

*where γ>1 is here a given integer.*

The rate of transition from state *u* to state *v* is proportional to the γ–th power of actual probability of state *v*. The higher is the probability, the larger is the chance of the change. The interaction kernel β corresponds to the tendency of agents to change a state. In particular, it may restrict the interactions only to states that are close each to other—see Ref. [16]. The (sensitivity) parameter γ describes the level of sensitivity of interactions: The greater is γ the more sensitive interactions are.

The models defined by Case 1 were proposed in [14], and then studied in various directions in [16,17,18]. Ref. [14] proposed results of global existence in the space homogeneous case for 0<γ<1, whereas γ>1 was considered in [16,17,18]. Assuming γ=1 for symmetric β yields a trivial model. Thus it was excluded as it is stated in Case 1. The detailed information on the modeling leading to Case 1 can be found in [19] (see also references therein), where it was referred to the conformist society.

We consider the following equation

(7) $$\frac{\partial }{\partial \;t}f\left(t,u\right)=Q\left[f\right]\left(t,u\right)\;t>0\;,\;u\in U\;,$$

with

(8) $$Q\left[f\right]\left(t,u\right)=\;f^{\gamma }\left(t,u\right)\int_{U}\beta \left(v,u\right)f\left(t,v\right)\;dv-f\left(t,u\right)\int_{U}\beta \left(u,v\right)f^{\gamma }\left(t,v\right)\;dv\;.$$

Independently we consider the following two, formally more general, kinetic equations

**Case** **2.** *Let*

$$\begin{array}{c} T\left[f\left(t,\;.\;\right)\right]\left(u,v\right)=\;\underset{\gamma \;\times }{\underset{︸}{\;\int_{U}\;\ldots \;\int_{U}}}A\left(v,u,v_{1}\;,\ldots \;,v_{\gamma }\right)\alpha \left(u,v_{1}\;,\ldots \;,v_{\gamma }\right)\;\times \\ f\left(t,v_{1}\right)\ldots f\left(t,v_{\gamma }\right)\;dv_{1}\;\ldots \;dv_{\gamma }\;,\;u,v\in U\;, \end{array}$$

*where γ is an integer.*

Case 2 leads to

(9) $$\frac{\partial }{\partial \;t}f\left(t,u\right)=\bar{Q}\left[f\right]\left(t,u\right)\;t>0\;,\;u\in U\;,$$

with

(10) $$\begin{array}{c} \bar{Q}\left[f\right]\left(t,u\right)=\underset{(\gamma +1)\;\times }{\underset{︸}{\;\int_{U}\;\ldots \;\int_{U}}}A\left(u,v,v_{1}\;,\ldots \;,v_{\gamma }\right)\alpha \left(v,v_{1},\;\ldots \;,v_{\gamma }\right)\;\times \\ f\left(t,v\right)f\left(t,v_{1}\right)\ldots f\left(t,v_{\gamma }\right)\;dv\;dv_{1}\;\ldots \;dv_{\gamma }\;-\\ f\left(t,u\right)\;\underset{\gamma \;\times }{\underset{︸}{\;\int_{U}\;\ldots \;\int_{U}}}\;\alpha \left(u,v_{1},\;\ldots \;,v_{\gamma }\right)\;f\left(t,v_{1}\right)\ldots f\left(t,v_{\gamma }\right)\;dv_{1}\;\ldots \;dv_{\gamma }\;. \end{array}$$

**Case** **3.** *Let γ be an integer and*

$$\begin{array}{c} T\left[f\left(t,\;.\;\right)\right]\left(u,v\right)=A_{0}\left(v,u\right)\alpha_{0}\left(v\right)f\left(t,v\right)\;+\\ +\;\sum_{j=1}^{\gamma }\;\underset{j\;\times }{\underset{︸}{\;\int_{U}\;\ldots \;\int_{U}}}A_{j}\left(v,u,v_{1},\;\ldots \;,v_{j}\right)\alpha_{j}\left(u,v_{1},\;\ldots \;,v_{j}\right)f\left(t,v_{1}\right)\ldots f\left(t,v_{j}\right)\;dv_{1}\;\ldots \;dv_{j}\;.\\ u,v\in U\;, \end{array}$$

Case 3 leads to

(11) $$\frac{\partial }{\partial \;t}f\left(t,u\right)=\tilde{Q}\left[f\right]\left(t,u\right)\;t\geq 0\;,\;u\in U\;,$$

with

(12) $$\begin{array}{c} \tilde{Q}\left[f\right]\left(t,u\right)=\;\int_{U}A_{0}\left(u,v\right)\alpha_{0}\left(v\right)f\left(t,v\right)\;dv-\alpha_{0}\left(u\right)f\left(t,u\right)\;+\\ +\;\sum_{j=1}^{\gamma }\;(\underset{(j+1)\;\times }{\underset{︸}{\;\int_{U}\;\ldots \;\int_{U}}}A_{j}\left(u,v,v_{1},\;\ldots \;,v_{j}\right)\alpha_{j}\left(v,v_{1},\;\ldots \;,v_{j}\right)\;\times \\ f\left(t,v\right)f\left(t,v_{1}\right)\ldots f\left(t,v_{j}\right)\;dv\;dv_{1}\;\ldots \;dv_{j}\;-\\ f\left(t,u\right)\;\underset{j\;\times }{\underset{︸}{\;\int_{U}\;\ldots \;\int_{U}}}\alpha_{j}\;\left(u,v_{1},\;\ldots \;,v_{j}\right)\;f\left(t,v_{1}\right)\ldots f\left(t,v_{j}\right)\;dv_{1}\;\ldots \;dv_{j})\;. \end{array}$$

The terms $A_{j}\left(u,v,v_{1},\ldots ,v_{j}\right)$ can be interpreted as the transition probabilities of changing from state *v* to *u* caused by interaction with agents with states $v_{1}$, $v_{2}$,...,$v_{j}$ whereas $a_{j}\left(v,v_{1},...,v_{j}\right)$ as rate of interaction between the agent with state *v* and agents with states $v_{1}$,...,$v_{j}$.

One may note that Equations (9) and (11), under suitable symmetry assumption, can be directly related with the dynamics of *N* interacting agents in the limit N→∞—see [8,13]. The former may be related to the interactions between γ agents, whereas the latter to interactions between *j* agents, with j=1,2,…,γ and j=0 corresponds to a stochastic change without any interaction—see [13]. One may note, however, that Equation (11) can be directly reduced to Equation (9) just taking $\alpha_{j}\equiv 0$ for each j=0,1,…,γ−1. On the other hand, thanks to the conservative properties, Equation (9) results in Equation (11) as well, under a suitable choice of *A* and α. For these reasons we concentrate on Equation (11) only.

The $L_{p}$-norm is denoted by ${∥\;.\;∥}_{p}$.

We may state the following local existence–uniqueness result for solutions to Equation (7).

**Proposition** **1.** *Let γ>1 and*

(13) $$\beta \in L_{\infty }\left(U\times U\right)\;.$$

*If $f_{0}$ is a probability density such that $f_{0}\in L_{\infty }\left(U\right)$, then there exists T>0 such that the solution f=f(t) to (7) exists and is unique in $L_{\infty }\left(U\right)\cap L_{1}\left(U\right)$ on the interval [0,T). The solution preserves positivity and $L_{1}$-norm (i.e., it is a probability density) on [0,T). Moreover,*

- *The solution, depending on initial data, is either global (T=∞) or local (T<∞).*
- *Under the additional assumption that β is a symmetric function—see [19]—the solution possesses all finite $L_{p}$-norms on [0,T), p>1, and the functions $t\mapsto {∥f\left(t\right)∥}_{p}$ are increasing for t∈[0,T).*

The first part of proof follows from [14]—see [19]—based on the Lipschitz property of the corresponding operator. The rest follows by *a priori* estimates.

From [16,17,18,19,20] we see that the behavior of the solution to Equation (7) may be very complex and may lead to various interesting applications in biology, medicine, and social sciences.

In contrast to Equation (7) with γ>1, Equation (7) with γ=1 (for asymmetric β), as well as Equations (9) and (11) result in the global existence–uniqueness of solutions.

**Proposition** **2.** *Let γ=1 and Equation (13) be satisfied. If $f_{0}$ is a probability density then for any T>0 the solution f=f(t) to (7) exists and is unique in $L_{1}\left(U\right)$ on the interval [0,T]. The solution preserves positivity and $L_{1}$-norm (i.e., it is a probability density) on [0,T].*

We consider the following conservative situation

**Assumption** **1.** *Let γ be an integer and*

(14) $$\begin{array}{c} A_{j}\geq 0\;,\;\alpha_{j}\geq 0\;,\;\alpha_{j}\in L_{\infty }\left(U^{j+1}\right)\;,\\ \int_{U}A_{j}\left(u,v,v_{1},\;\ldots \;,v_{j}\right)\;du=1\;\mathrm{for}\;\mathrm{all}\;\left(v,v_{1},\;\ldots \;,v_{j}\right)\in U^{j+1}\\ \mathrm{such}\;\mathrm{that}\;\alpha_{j}\left(v,v_{1},\;\ldots \;,v_{j}\right)>0\;,\;\forall \;j=1,\ldots ,\gamma \;. \end{array}$$

**Proposition** **3.** *Let Assumption 1 be satisfied. If $f_{0}$ is a probability density then for any T>0 the solution f=f(t) to Equation (11) exists and is unique in $L_{1}\left(U\right)$ on the interval [0,T]. The solution preserves positivity and $L_{1}$-norm (i.e., it is a probability density) on [0,T].*

**Corollary** **1.** *The solutions in Propositions 2 and 3 are in $L_{\infty }\left(U\right)$ on every compact [0,T] provided that $f_{0}\in L_{\infty }\left(U\right)$.*

The proofs of Propositions 2 and 3 are standard and based on the Lipschitz property in $L_{1}\left(U\right)$—cf. [20]. Similarly Corollary 1 follows.

Moreover, we need the smoothness of the solutions. Let $W^{m,p}\left(U\right)$ and $C_{B}^{m}\left(U\right)$ be the Banach spaces—the classical Sobolev space (a subspace of $L_{p}\left(U\right)$) and the space of *m*–differentiable functions with the usual norms denoted by ${∥\;.\;∥}_{p}^{\left(m\right)}$ and ${∥\;.\;∥}_{\left[B\right]}^{\left(m\right)}$, respectively—see [21].

Let $X^{\left(m\right)}=W^{m,1}\left(U\right)\cap C_{B}^{m}\left(U\right)$, m=0,1,2,…, and ${∥\;.\;∥}^{\left(m\right)}$ be defined

$${∥\;.\;∥}^{\left(m\right)}={∥\;.\;∥}_{p}^{\left(m\right)}+{∥\;.\;∥}_{\left[B\right]}^{\left(m\right)}\;,\;m=0,1,2,\ldots \;.$$

In particular, for m=0, we write $X=X^{\left(0\right)}=L_{1}\left(U\right)\cap L_{\infty }\left(U\right)$ and $∥\;.\;∥={∥\;.\;∥}^{\left(0\right)}$.

**Proposition** **4.** *Let the assumption of Proposition 1 be satisfied and additionally $f_{0}\in X^{\left(m\right)}$ and*

(15) $$\int_{U}\;\beta \left(u,v\right)g\left(v\right)\;dv\in X^{\left(m\right)}\;\mathrm{for}\;\mathrm{each}\;g\in X^{\left(m\right)}\;,$$

*for some m=1,2,3,…. Then the solution f=f(t) (given by Proposition 1) satisfies $f\left(t,\;.\;\right)\in X^{\left(m\right)}$ for all t∈[0,T).*

## 4. Macroscopic Behavior in the Conservative Case

In the present section we fix our attention on the behavior of the cumulative distribution function corresponding to the solution of a (mesoscopic) kinetic equation.

For simplicity we assume that $U=[\;0,\infty \;[\;\equiv R_{+}^{1}$ and γ=3, however possible generalizations are straightforward.

We show that, for particular assumptions on the parameters $A_{j}$, $\alpha_{j}$, j≤3, of Equation (11), the solution f=f(t,u) leads to the distribution

(16) $$F\left(t,u\right)=\int_{0}^{u}f\left(t,\tilde{u}\right)\;d\tilde{u}\;,$$

that possesses a diauxic growth with respect to t>0, for any sufficiently large u>0.

**Assumption** **2.** *We assume the interactions such that*

(17) $$\alpha_{j}\left(u,v_{1},\;\ldots \;,v_{j}\right)=j!\;\eta_{j}\;\chi \left(v_{1}\leq u\right)\chi \left(v_{2}\leq v_{1}\right)\;\cdots \;\chi \left(v_{j}\leq v_{j-1}\right)\;\mathrm{for}\;\mathrm{all}\;u,v_{1},\;\ldots \;,v_{j}\in R_{+}^{1}\;,$$

*where $\chi \left(\mathrm{true}\right)=1$, $\chi \left(\mathrm{false}\right)=0$, $\eta_{j}$ are positive constants,*

(18) $$\int_{0}^{u}A_{j}\left(\tilde{u},v,w_{1},\;\ldots \;,w_{j}\right)\;d\tilde{u}=\chi \left(w_{1}\leq u\right)\;\mathrm{for}\;\mathrm{all}\;u,v,w_{1},\;\ldots \;,w_{j}\in R_{+}^{1}\;,$$

*j=1,2,3 (we keep in mind that γ=3), with the standard convention, i.e., if j=1, then $w_{1},\;\ldots \;,w_{j}$ means $w_{1}$, if j=2, then $w_{1},\;\ldots \;,w_{j}$ means $w_{1},w_{2}$, if j=3, then $w_{1},\;\ldots \;,w_{j}$ means $w_{1},w_{2},w_{3}$, and*

(19) $$\alpha_{0}\left(u\right)=\eta_{0}\;\mathrm{for}\;\mathrm{any}\;u\in R_{+}^{1}\;,$$

(20) $$\int_{0}^{u}A_{0}\left(\tilde{u},v\right)\;d\tilde{u}=\zeta \left(u\right)\;\mathrm{for}\;\mathrm{all}\;u\geq u_{0}\;\mathrm{and}\;v\in R_{+}^{1}\;,$$

*where $u_{0}>0$ is a given constant and ζ is a increasing function such that ζ(0)=0 and $\lim_{u\to \infty }\zeta \left(u\right)=1$.*

We may note, that Assumption 2 implies Assumption 1.

By Equation (17) and simple calculations, we obtain

(21) $$\begin{array}{c} \int_{0}^{u}\underset{j\;\times }{\underset{︸}{\;\overset{\infty }{\underset{0}{\int }}\;\ldots \;\overset{\infty }{\underset{0}{\int }}}}\;f\left(t,\tilde{u}\right)\;\alpha_{j}\;\left(u,v_{1},\;\ldots \;,v_{j}\right)\;f\left(t,v_{1}\right)\ldots f\left(t,v_{j}\right)\;dv_{1}\;\ldots \;dv_{j}\;d\tilde{u}\;=\\ \frac{\eta_{j}}{j+1}\;{\left(\int_{0}^{u}f\left(t,\tilde{u}\right)\;d\tilde{u}\right)}^{j+1}\;, \end{array}$$

for *j* equal 1, 2 and 3, and any $f\left(t,\cdot \right)\in L_{1}\left(R_{+}^{1}\right)$ and

(22) $$\int_{0}^{u}\;f\left(t,\tilde{u}\right)\;\alpha_{0}\left(\tilde{u}\right)\;d\tilde{u}=\eta_{0}\;\int_{0}^{u}f\left(t,\tilde{u}\right)\;d\tilde{u}\;.$$

Moreover, for any $f\left(t,\cdot \right)\in L_{1}\left(R_{+}^{1}\right)$ such that ${∥f∥}_{1}=1$, j=1,2,3, by Equations (17) and (18), we have

(23) $$\begin{array}{c} \int_{0}^{u}\;\underset{(j+1)\;\times }{\underset{︸}{\;\overset{\infty }{\underset{0}{\int }}\;\ldots \;\overset{\infty }{\underset{0}{\int }}}}A_{j}\left(\tilde{u},v,v_{1},\;\ldots \;,v_{j}\right)\alpha_{j}\left(v,v_{1},\;\ldots \;,v_{j}\right)\;\times \\ f\left(t,v\right)f\left(t,v_{1}\right)\ldots f\left(t,v_{j}\right)\;dv\;dv_{1}\;\ldots \;dv_{j}\;d\tilde{u}\;=\\ j!\;\eta_{j}\;\int_{0}^{\infty }f\left(t,v\right)\int_{0}^{\infty }f\left(t,v_{1}\right)\chi \left(v_{1}\leq v\right)\chi \left(v_{1}\leq u\right)\int_{0}^{v_{1}}f\left(t,v_{2}\right)\;\ldots \int_{0}^{v_{j-1}}f\left(t,v_{j}\right)\;dv\;dv_{1}\;\ldots \;dv_{j}\;d\tilde{u}\;=\\ I_{1}+I_{2}\;, \end{array}$$

where

$$I_{1}=\frac{\eta_{j}}{j+1}\;{\left(\int_{0}^{u}f\left(t,\tilde{u}\right)\;d\tilde{u}\right)}^{j+1}\;,$$

and

$$I_{2}=\eta_{j}\left({\left(\int_{0}^{u}f\left(t,\tilde{u}\right)\;d\tilde{u}\right)}^{j}-{\left(\int_{0}^{u}f\left(t,\tilde{u}\right)\;d\tilde{u}\right)}^{j+1}\right)\;.$$

Finally, for any $f\in L_{1}\left(R_{+}^{1}\right)$ such that ${∥f∥}_{1}=1$, by Equations (19) and (20), for any $u>u_{0}$ we have

(24) $$\int_{0}^{u}\;\int_{0}^{\infty }A_{0}\left(\tilde{u},v\right)\alpha_{0}\left(v\right)f\left(t,v\right)\;dv\;d\tilde{u}=\eta_{0}\;\zeta \left(u\right)\;.$$

By the above calculations, integrating Equation (11) with respect to *u*, we can see that any solution *f* of Equation (11), corresponding to an initial datum that is a probability density, is such that x(t)=F(t,u) given by Equation (16), for any fixed $u>u_{0}$, satisfies the following equation

(25) $$\dot{x}=W\left(x\right)\;,$$

where

(26) $$W\left(x\right)=-\eta_{3}x^{4}+\left(\eta_{3}-\eta_{2}\right)x^{3}+\left(\eta_{2}-\eta_{1}\right)x^{2}+\left(\eta_{1}-\eta_{0}\right)x+\eta_{0}\zeta \left(u\right)\;,$$

where *u* is treated here as a (fixed) parameter.

Therefore, it is easy to see that the parameters of the model can be chosen in such a way that t→F(t,u) possesses a diauxic growth for any fixed sufficiently large *u*. We then obtain

**Corollary** **2.** *Let Assumption 2 be satisfied and $f_{0}$ be a probability density on $U=R_{+}^{1}$. The solution f=f(t,u) to Equation (11), given by Proposition 3, is such that the corresponding F=F(t,u) given by Equation (16) has a diauxic growth with respect to t, for any sufficiently large $u\in R_{+}^{1}$.*

## 5. Macroscopic Behavior in the Nonconservative Case

In order to adapt to a situation typical in game theory—cf. Section 2, we replace Assumption 1 by the following more general statement.

**Assumption** **3.** *Let γ be an integer and*

(27) $$\begin{array}{c} A_{j}\geq 0\;,\;\alpha_{j}\geq 0\;,\;\alpha_{j}\in L_{\infty }\left(U^{j+1}\right)\;,\\ A_{j}\left(\;.\;,v,v_{1},\;\ldots \;,v_{j}\right)\in L_{1}\left(U\right)\;\mathrm{for}\;\mathrm{all}\;\left(v,v_{1},\;\ldots \;,v_{j}\right)\in U^{j+1}\\ \mathrm{such}\;\mathrm{that}\;\alpha_{j}\left(v,v_{1},\;\ldots \;,v_{j}\right)>0\;,\;\forall \;j=0,\ldots ,\gamma \;. \end{array}$$

In this section, we deal with the macroscopic behavior derived by the mesoscopic structures defined in the previous section.

We decompose $U=U_{*}\cup U^{*}$, where $U_{*}$ and $U^{*}$ are arbitrary (Lebesgue) measurable sets such that $U_{*}\cap U^{*}=\emptyset$, both with positive (Lebesgue) measures. For a given solution *f* of the mesoscopic equation we are interested in the behavior of

(28) $$\int_{U_{*}}f\left(t,v\right)\;dv\;\mathrm{and}\;\int_{U^{*}}f\left(t,v\right)\;dv$$

that can be related to μ(t) and ν(t), cf. Equation (4), as well as

$$\frac{\int_{U_{*}}f\left(t,v\right)\;dv}{\int_{U}f\left(t,v\right)\;dv}$$

that can be related to x(t) in the macroscopic description, cf. Equation (1) with Equation (5).

Similarly to that of [22], we assume a direct dependence of the rate $\alpha_{2}$ on the unknown function *f* in Equation (11). This is a Enskog-type of assumption known in kinetic theory—cf. [23] and references therein.

**Assumption** **4.** *We assume*

1. *$\alpha_{0}=\alpha_{1}$ and*
  $$\alpha_{2}=\alpha_{2}\left(f\left(t\right);v_{1},v_{2},v_{3}\right)=\frac{\kappa }{{\left(\int_{U}f\left(t,u\right)\;du\right)}^{2}}\;,$$
2. *$A_{2}=A_{2}\left(u,v_{1},v_{2},v_{3}\right)$ is such that*
  *(a)*  $\int_{U_{*}}A\left(u,v_{1},v_{2},v_{3}\right)\;du=\frac{a_{1}}{\kappa }$, if $v_{1},v_{2},v_{3}\in U_{*}$;
  *(b)*  $\int_{U_{*}}A\left(u,v_{1},v_{2},v_{3}\right)\;du=\frac{2\;a_{2}}{3\;\kappa }$, if $v_{i}\in U^{*}$, for some i=1,2,3, and $v_{j}\in U_{*}$ for each j=1,2,3 such that j≠i;
  *(c)*  $\int_{U_{*}}A\left(u,v_{1},v_{2},v_{3}\right)\;du=\frac{a_{3}}{3\;\kappa }$, if $v_{i}\in U_{*}$, for some i=1,2,3, and $v_{j}\in U^{*}$ for each j=1,2,3 such that j≠i;
  *(d)*  $\int_{U^{*}}A\left(u,v_{1},v_{2},v_{3}\right)\;du=\frac{b_{1}}{3\;\kappa }$, if $v_{i}\in U^{*}$, for some i=1,2,3, and $v_{j}\in U_{*}$ for each j=1,2,3 such that j≠i;
  *(e)*  $\int_{U^{*}}A\left(u,v_{1},v_{2},v_{3}\right)\;du=\frac{2\;b_{2}}{3\;\kappa }$, if $v_{i}\in U_{*}$, for some i=1,2,3, and $v_{j}\in U^{*}$ for each j=1,2,3 such that j≠i;
  *(f)*  $\int_{U^{*}}A\left(u,v,v_{1},v_{2}\right)\;du=\frac{b_{3}}{\kappa }$, if $v,v_{1},v_{2}\in U^{*}$.

Assume now that the payoffs $a_{1},a_{2},a_{3},b_{1},b_{2},b_{3}$, see Section 2, are such that the corresponding Equation (1) with Equation (5) result in solutions that have a diauxic growth—cf. [7]. Then the kinetic Equation (11) leads to diauxic growth of (28) if Assumption 4 is satisfied. In fact

**Theorem** **1.** *Let Assumption 4 be satisfied and $f_{0}\in L_{1}\left(U\right)$ be nonnegative and such that*

$$\int_{U_{*}}f_{0}\left(u\right)\;du>0\;.$$

*Then, for any t>0, there exists a unique solution f=f(t) of Equation (11) in $L_{1}\left(U\right)$. Moreover it is possible to choose the payoffs $a_{1},a_{2},a_{3},b_{1},b_{2},b_{3}$ in such a way that (28) given by the solution f=f(t) has a diauxic growth.*

**Proof.**  It is standard to see that the operator defined by the right-hand-side of Equation (11) is locally Lipschitz continuous in $L_{1}\left(U\right)$. Then a local in time solution f=f(t) exists in $L_{1}\left(U\right)$ and it is unique. It is also standard that the solution preserves nonnegativity of the initial datum. We observe that $\mu \left(t\right):=\int_{U_{*}}f\left(t,u\right)\;du$ and $\nu \left(t\right):=\int_{U^{*}}f\left(t,u\right)\;du$ satisfy Equation (4) on the interval of time of existence of the solution. Therefore $\frac{\mu (t)}{\mu (t)+\nu (t)}$ satisfies Equation (1) on the same time interval. By the form of Equation (4), we observe that any solution of Equation (4) must be bounded on any compact interval. This delivers an *a priori* estimate of the $L_{1}\left(U\right)$-norm of the solution, which concludes the proof. □

**Remark** **1.** *For simplicity, we assumed at the beginning that all payoffs were nonnegative. It is easy to see that Assumption 4 can be easily modified to cover the case if any of payoffs is negative.*

## 6. Concluding Remarks

In the paper, we show that some mesoscopic models can produce a diauxic behavior on the macroscopic level. In such a case, the macroscopic picture is more complex that the usual one of a logistic-type, similar to the curve of cumulative normal distribution function (and thus related to the central limit theorem) with only one inflection point. The paper should be understood as the first step of description the relationships between the mesoscopic and macroscopic scales where new and interesting effects can appear. One may hypothesize that a complex but organized behavior on the level of micro-scale or meso-scale can lead to the diauxic macroscopic growth. This, however, still needs a new mathematical background.
